# Cessation of Deliberate Self-Harm Behavior in Patients With Borderline Personality Traits Treated With Outpatient Dialectical Behavior Therapy

**DOI:** 10.3389/fpsyg.2021.578230

**Published:** 2021-02-26

**Authors:** Yngvill Ane Stokke Westad, Kristen Hagen, Egil Jonsbu, Stian Solem

**Affiliations:** ^1^Helse Møre og Romsdal Hospital Trust, Molde, Norway; ^2^Department of Mental Health, Faculty of Medicine and Health Sciences, Norwegian University of Science and Technology, Trondheim, Norway; ^3^Department of Psychology, Norwegian University of Science and Technology, Trondheim, Norway

**Keywords:** self-harm, borderline personality disorder, dialectic behavioral therapy, DBT, suicide

## Abstract

The first aim of the study was to identify when deliberate self-harm (DSH) behavior ceased in patients with borderline symptoms undergoing dialectical behavioral treatment (DBT). The second aim was to compare patients who ceased their self-harm behavior early or late in the course of treatment, with regard to demographics, comorbidity, and symptom severity. The study used a naturalistic design and included 75 treatment completers at an outpatient DBT clinic. Of these 75 patients, 46 presented with self-harming behavior at pre-treatment. These 46 participants where split into two groups, based on median amount of time before ceasing self-harm behavior, termed early (up to 8 weeks) and late (8+ weeks) responders. Treatment duration varied from 16 to 160 weeks. Patients were assessed pre- and post-treatment using measures of depression, hopelessness, personality traits, quality of life, and global assessment of symptoms and functioning. The majority (93.5%) ceased their self-harming within the first year, and the average number of weeks was 15.5 (*SD* = 17.8). Twenty-five percent of patients ceased their DSH behavior during the first week of treatment. For the remaining patients, the cessation of DSH continued gradually across a 1 year period. We found no differences between early and late responders with respect to demographics, comorbidity, symptom severity, or treatment outcome. None of the patients committed suicide. The findings indicate that self-harming behavior decreases gradually across the first year after starting DBT.

## Introduction

Deliberate self-harm behavior (DSH) is a transdiagnostic phenomenon, occurring across a range of psychiatric disorders (Selby et al., 2012). The behavior also occurs in non-clinical samples (Levine et al., 2020). Self-harming behavior is often repeated (Owens et al., 2002), and is one of the most frequent reasons for emergency hospital admission (Carroll et al., 2014). Studies have consistently shown that manifestation of self-harm behavior is a known risk factor for suicide (Owens et al., 2002; Urnes, 2009; Asarnow et al., 2011; Whitlock et al., 2013; Hawton et al., 2015). The presence of DSH therefore poses a considerable burden on health care services (Hawton and Sinclair, 2003; Sinclair et al., 2011).

One of the psychiatric diagnoses strongly associated with deliberate self-harm is borderline personality disorder (BPD), were the presence of DSH is one of the diagnostic criteria for the disorder (Selby et al., 2012). Between 50 and 80% of patients with BPD present deliberate self-harm behavior (Oumaya et al., 2008). Being especially prone to suicidal behavior, the average patient with BPD attempts suicide 3.3 times and up to 10% completes suicide (Black et al., 2004; Zanarini et al., 2008; McMain et al., 2018). The presence of DSH in BPD complicates the clinical assessment of suicidality, and may lead to inexpedient psychiatric hospitalizations and therapist burn-out which, at worst, could deteriorate long-term treatment prognosis (Mehlum and Jensen, 2006; van den Bosch et al., 2014). This necessitates the implementation of effective treatment that improves patients’ self-harming behavior.

Several psychotherapies are considered effective for borderline personality disorder (BPD) (Cristea et al., 2017). One of the most recognized treatment interventions to treat suicidal-, self-harm- and other destructive behaviors commonly observed among patients with BPD is Dialectic Behavioral Therapy (DBT). DBT is a method developed for patients with BPD showing serious suicidal and self-harm behaviors, and is explicitly aimed at quickly gaining control of self-harm and other types of life-threatening behaviors (Linehan, 1993). A number of randomized controlled studies have provided empirical support for its efficiency (Priebe et al., 2012; Stoffers et al., 2012; DeCou et al., 2019), and meta-analyzes of treatment has indicated a moderate effect size for suicidal- and self-harming behavior in patients with BPD (Kliem et al., 2010).

Although DBT has a documented effect in reducing DSH in patients with BPD, knowledge of the trajectories of self-harm, and exactly when in the course of treatment DSH may finally cease, is still sparse. After conducting a systematic research on evaluating the effects of different psychological intervention for people with borderline personality disorder, Binks et al. (2006) concluded that self-harm or parasuicide may decrease at 6–12 months. In an adolescent population comparing DBT with individual and group supportive therapy (IGST), McCauley et al. (2018) found that 46.6% did no longer present self-harm after 6 months (treatment ending) of DBT treatment (vs. 27.6% in IGST). At 6-month follow-up, the rates had increased to 51.2% in DBT (vs. 32.2% in IGST). Even though self-harm behavior decreases during DBT, van Goethem et al. (2015) found evidence for a highly variable and erratic course of parasuicidal behavior during treatment in an adult BPD sample. In the 13 patients they followed, they noted a considerable high between-patient difference in the course of self-harm behavior. At follow-up, 27% still presented with self-injurious behavior. Further there are some indications for more changes in self-injurious behavior during the first half year of DBT treatment than in the second half year (van Goethem et al., 2012). Three treatment trajectories have been suggested: a rapid response group, a slow progression group, and a relapse group (McMain et al., 2018). Further knowledge about subgroup trajectories could inform possible tailoring of individualized treatment interventions.

The main purpose of this study was therefore to investigate when adult patients with borderline symptoms ceases their deliberate self-harm behavior. The second aim was to compare patients who early or late in the course of treatment ceased their deliberate self-harm behavior. These two groups are compared with respect to age, gender, comorbidity, symptoms of depression, hopelessness, quality of life, personality pathology, global functioning and symptoms, and suicide rate.

## Materials and Methods

### Participants and Procedure

A naturalistic design was used. All patients referred to the regional Dialectical Behavior Therapy (DBT) outpatient community mental health clinic for adults in the county of Møre and Romsdal (Norway) in the time period from 2007 to 2014 were included. Patients were referred either from other mental health services, from somatic hospitals, or from GPs. After referral, patients considered likely to present borderline symptomatology were invited to the DBT clinic to get an orientation to treatment (2–3 appointments). During orientation, motivation to commit to therapy were addressed (i.e., willingness to build “a life worth living,” filling out weekly diary card, trying out different DBT skills, consented with the DBT treatment hierarchy etc.). Patients, which at the time of orientation confirmed borderline symptoms, and could commit to DBT treatment, went through a diagnostic evaluation and were accepted into the DBT treatment program when goals of treatment were defined.

The inclusion criteria were: meeting criteria or sub-threshold criteria for borderline personality disorder, above 18 years, speak Scandinavian, and willing to commit to DBT treatment. The exclusion criteria were: the patient fulfilled the diagnostic criteria as stated in the DSM-IV (American Psychiatric Association., 2013) for: antisocial personality disorder, major depressive disorder, active psychosis, ongoing drug misuse/dependence as a primary problem, intellectual disability, or a Body Mass Index (BMI) below 17.

**FIGURE 1:**
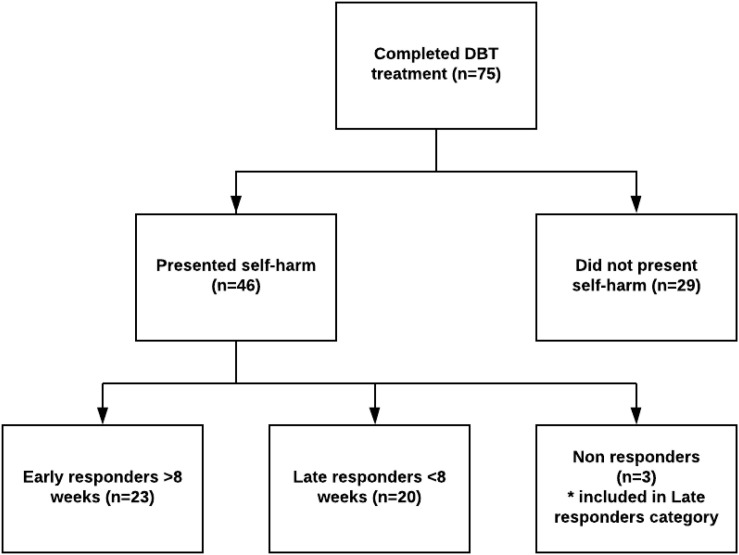
Flow of DBT treatment completers in regard to DSH behavior cessation. Pre- and post-treatment data for all study variables were available for all patients presenting with self-harm.

Figure 1 shows the participant flow of DBT treatment completers with regard to DSH behavior cessation. Only patients that presented DSH and completed DBT treatment were included in statistical analyzes. The total number of patients meeting to DBT orientation in the designated time period were 154. Seventy-five of these patients completed DBT treatment. Patients who did not complete treatment either met exclusion criteria, did not meet inclusion criteria or were not motivated to DBT treatment at the phase of orientation, moved away during therapy, were referred to other health care services, or dropped out of therapy prematurely.

The DSH patients were split into two groups, based on median amount of time (8 weeks) in DBT before successfully ceasing their DSH behavior, termed “early” and “late” responders. Three patients did not cease their DSH during treatment. These patients were too few to analyze separately, and were therefore included in the “late responders” category. This left 23 patients classified as early responders and 23 late responders.

Of the 75 treatment completers, 46 (61%) reported engaging in DSH at treatment baseline. The majority of these DSH presenting patients were women (91.3%) and the mean age was 24 (range 18–45). About half (55.6%) of the patients fulfilled the criteria for a BPD diagnosis (as opposed to sub-threshold BPD). The average number comorbid axis-I diagnosis were 2.6 (range 0–6). The majority of patients used cutting as preferred method for self-harm (*n* = 41, 89%). Other self-harm methods included hitting themselves in head or hitting their hands on something (*n* = 7), scratching (*n* = 1), burning (*n* = 1), biting themselves (*n* = 1), chewing in the oral cavity (*n* = 1), or causing damage to the genital area (*n* = 1).

### Treatment

DBT is a multicomponent cognitive-behavioral treatment. The standard treatment consists of a combination of weekly individual therapy (1 h), participation in skill groups (2.5 h), skills training, telephone consultation and a consulting team for DBT therapist (Linehan and Wilks, 2015). After completing the skill group, the patient could continue in individual therapy if the consultation team expected benefit from further follow-up. A new round in skill group could be granted if the consultation team considered it favorable for treatment outcome. The average duration of treatment was 61.6 weeks (*SD* = 32.9). In comparison the meta-analysis of Kliem et al. (2010) found a range of 12–52 weeks for DBT trials with an average duration of 40 weeks (*SD* = 17.2). They also found that duration did not moderate treatment effect.

All DBT therapists worked both as individual therapists and as skill group trainers. The therapists were either certified DBT therapists, or in a DBT certification program. All therapists participated in the weekly consultation team. Treatment sessions were recorded on video and a selection of these were reviewed both in consultation team and by an expert DBT supervisor to ensure the competence and adherence of the treatment.

### Diagnostics

Personality traits were assessed prior to initiation of treatment with the Personality Diagnostic Questionnaire—4th Edition Plus (PDQ-4+; Hyler, 1994). The personality traits of those participants that scored above clinical cut-off (either on total score and/or on the borderline personality subcategory) of the PDQ-4 where further assessed with the Structured Clinical Interview for DSM-4 Axis-II Personality Disorders (Spitzer et al., 1990). Symptom diagnoses were evaluated administrating the MINI International Neuropsychiatric Interview (Sheehan et al., 1998). Final diagnoses were discussed in a consultation team with an experienced clinician.

### Primary Outcome Measures

In DBT the presence of self-harm and suicide behavior are top priority in the treatment hierarchy. As a DBT therapy strategy, the occurrence of any self-harm or suicide behavior is to be addressed at the following therapy appointment. The presence of self-harm were registered in weekly medical records based on patients daily registration of the occurrence of self-harm on their diary chart, optimizing the quality assurance of the outcome variable. Deliberate self-harm was defined as deliberate, direct destruction of body tissue without conscious suicidal intent, but resulting in injury severe enough for tissue damage (e.g., scarring) to occur. A systematic review of the medical records were done by the first author to collect the occurrence, preferred method, duration and cessation of DSH behavior and instances of suicide. The number of weeks from treatment baseline until DSH did no longer occur were recorded. All records were throughout reviewed to make sure the DSH did not reoccur at a later stage of treatment.

### Secondary Outcome Measurements

The severity of depression symptoms was measured with the Beck Depression Inventory (BDI; Beck et al., 1961). BDI consists of 21 groups of statements. Each group has four response options with scoring width from 0 (does not confirm the experience) to 4 (confirms the full experience). Higher scores indicate an increasing severity in symptoms of depression. The BDI has shown good psychometric properties (Beck et al., 1988).

Experienced hopelessness was assessed with Becks Hopelessness Scale (BHS; Beck and Ster, 1988). The scale consists of 20 statements, each answered with “correct” or “wrong.” Higher scores indicate an increased sense of hopelessness. The BHS has good psychometric properties (Bouvard et al., 1992).

The personality Diagnostic Questionnaire—4th Edition Plus (PDQ-4+; Hyler, 1994) is a 99-item true-false questionnaire that yields personality diagnoses consistent with the DSM-IV. The PDQ-4+ yields diagnostic criteria for the 10 personality disorders in DSM-IV in addition to two other personality disorders (passive-aggressive and depressive personality). A total score of 30 or more indicate a likelihood of presenting personality disturbance (Bagby and Farvolden, 2004). Davison et al. (2001) concluded that the PDQ-4 + appears to has properties suitable for use as a screening instrument for the presence or absence of personality disorders.

Quality of life was assessed with the World Health Organization Quality of Life Scale –Abbreviated form (WHOQOL-BREF; WHOQOL Group, 1998). The instrument consists of 26 questions where two of which measures quality of life and general health overall. The rest measures four life quality domains; physical, psychological, social relationships and environmental. Items are scored on a scale from 1 to 5. The scores are further transformed into a linear scale ranging from 0 to 100, where higher scores indicates increased sense of life quality in the respective domains. In this study, only the score for overall quality of life was used as an outcome variable. The WHOQOL-BREF has shown good to excellent psychometric properties of reliability, and well in preliminary tests of validity (Skevington et al., 2004).

The Global Assessment of function scale (GAF) is the Axis-V of the Diagnostic and Statistical Manual of Mental Disorders, Fourth Edition (DSM-IV-TR; American Psychiatric Association., 2000). GAF is an observer-rated overall measure of how patients are doing. It measures psychological, social and occupational functioning, covering a range score from 0 to 100. Lower scores indicate higher symptom severity and lower function levels. GAF values can be either a single score or separate scores for symptoms (GAF-S) and functioning (GAF-F) (Aas, 2011). A score of 60 represents a cut-off level between mild/no impairment and moderate/severe impairment (Kvarstein et al., 2019). The split version was used in this study.

### Statistics

In order to investigate possible differences between the early and late responder groups at pre- and post-treatment, independent *t*-tests were conducted. To explore possible changes- and interaction effects in symptoms from pre- to post-treatment, split plot (early vs. late responders) repeated measures ANOVAs were conducted. In order to allow participants with missing data to be included in the analyses, missing data were replaced using the expectation maximization (EM) method of SPSS, version 24. The expectation maximization algorithm can be used when less than 25% of a data set is missing and the data are missing at random, which was the case for this data set [Little’s MCAR test χ^2^(125) = 120.23, *p* = 0.604].

### Ethics

All patients were informed of systematic data collection for the purpose of research and quality assurance. All participants had signed a written consent at the time of initiation of treatment. They were also informed that non-agreement would not affect the offer of current or future treatment propositions. None of the patients offered to participate in this study refused participation. Ethical approval was obtained from the regional committees for medical and health research ethics (ref.nr. 2015/1485).

## Results

**FIGURE 2:**
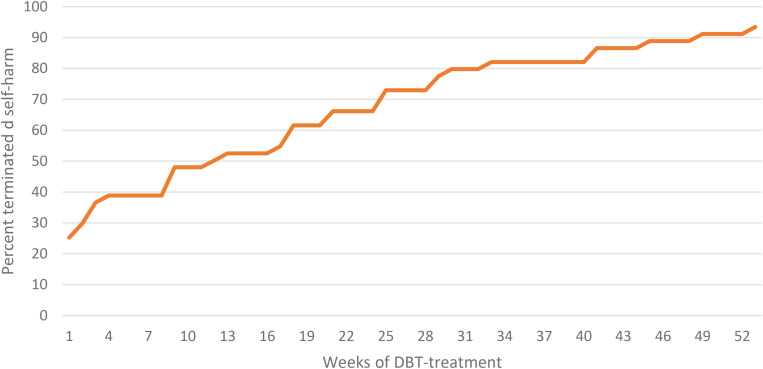
Cumulative percentage of patients that terminated self-harm behavior in the treatment period.

During DBT treatment, 93.5% of the participants ceased their DSH behavior within the first year after starting treatment. Three patients (6.5%) continued to present DSH throughout the treatment period. The patients that ceased their self-harm behavior used on average 15.5 weeks (*SD* = 17.8) to end this behavior. The cumulative percent that successfully ceased the self-harming behavior is presented in Figure 2. None of the participants in either groups committed suicide during treatment.

We found no significant difference between early and late responders on demographic variables. Neither were there any significant differences between groups on diagnostic characteristics (number of comorbid disorders, percentage that reached full BPD diagnosis contrary to subthreshold BPD). See Table 1 for details.

**TABLE 1:** Baseline demographics and clinical characteristics for patients ending the self-harm early (before 8 weeks) and late (after 8 weeks).

	Early responders (*n* = 23) M (SD)/n (%)	Late responders (*n* = 23) M (SD)/n (%)	*p*
Age	23.00 (5.74)	25.04 (8.39)	0.34
Female sex	21 (91.30)	21 (91.30)	1.00
BPD	13 (56.52)	13 (56.52)	1.00
Number of comorbid axis-II disorders	1.08 (1.05)	1.17 (0.78)	0.75
Number of comorbid axis-I disorders	2.23 (1.29)	2.87 (1.29)	0.14
Treatment duration (weeks)	55.65 (28.64)	67.65 (36.34)	0.22
Weeks before ending self-harm	1.87 (3.00)	27.15 (12.03)	< 0.01
**Comorbid disorders**			
GAD	4 (17.4)	6 (26.1)	
Eating disorder, unspecified	9 (39.1)	12 (52.2)	
Bulimia nervosa	2 (8.7)	0 (0.0)	
OCD	1 (4.4)	3 (13.0)	
Depression	9 (39.1)	11 (47.8)	
PTSD	4 (17.4)	4 (17.4)	
Dysthymia	7 (30.4)	8 (34.8)	
Hypochondria	2 (8.7)	0 (0.0)	
Social anxiety disorder	4 (17.4)	8 (34.8)	
Agoraphobia	4 (17.4)	8 (34.8)	
Specific phobia	1 (4.4)	2 (8.7)	
Alcohol/drug disorder	1 (4.4)	3 (13.0)	
Insomnia	0 (0.0)	1 (4.4)	
Mixed anxiety and depressive disorder	0 (0.0)	1 (4.4)	
Adjustment disorder	0 (0.0)	1 (4.4)	

*BPD, Diagnosed with Borderline personality disorder; GAD, Generalized anxiety disorder; OCD, Obsessive-compulsive disorder; PTSD, Post-traumatic stress disorder.*

On clinical characteristics, we found no differences between the early and late responders on depression (BDI), hopelessness (BHS), personality disorders (PDQ-4), Quality of life (WHOQOL-BREF), or global function (GAF-F) and symptoms (GAF-S) at pre-treatment. The same pattern was evident after treatment, as there were no significant differences between the early- and late responders. There were significant improvements in BDI, BHS, PDQ-4, WHOQOL-BREF, GAF-F, and GAF-S scores from pre- to post-treatment. However, there were no interaction effects between time and group (early vs. late responders). See Table 2 for details.

**TABLE 2:** Means and standard deviations for BDI, BHS, PDQ-4, WHOQOL-BREF, and GAF before and after treatment.

	Pre-treatment		Post-treatment		*Time*		*Time x group*	
	Early responders	Late responders	Early responders	Late responders	*F*	η*_*p*_^2^*	*F*	η*_*p*_^2^*
BDI	31.87 (10.53)	28.35 (8.54)	13.64 (9.77)	13.74 (9.74)	83.7*	0.66	1.0	0.02
BHS	13.49 (4.60)	12.27 (4.82)	5.55 (4.79)	6.52 (4.93)	81.2*	0.65	2.1	0.05
PDQ-4	46.04 (12.42)	41.39 (10.59)	27.35 (13.37)	30.25 (13.81)	52.6*	0.54	0.2	0.00
WHOQOL-BREF	2.29 (0.88)	2.43 (0.83)	3.54 (0.78)	3.35 (0.83)	52.6*	0.55	1.3	0.03
GAF-F	51.78 (6.52)	54.39 (11.34)	69.43 (12.04)	68.65 (10.81)	119.1*	0.73	1.3	0.03
GAF-S	50.78 (4.97)	54.39 (6.88)	70.12 (11.47)	66.57 (10.37)	134.1*	0.75	3.8	0.08

BDI, Beck depression Inventory; BHS, Beck Hopelessness Inventory; PDQ-4, Personality Diagnostic Questionnaire for DSM 4; WHOQOL-BREF, World Health Organization quality of Life Scale; GAF-F, Global Assessment of Functioning—Function; GAF-S, Global Assessment of Functioning—Symptoms. *p < 0.001.

A comparison between patients with self-harm and those without, revealed no significant differences on BDI, BHS, PDQ-4, and GAF at pre- and post-treatment. However, there was a significant age difference between the two groups as the self-harm group was younger, 30.55 years (11.18) vs. 24.02 (7.18), *t* = 2.80, *p* = 0.008. There were no significant differences in treatment length or number of diagnoses for the two groups.

## Discussion

On average, it took 15.5 weeks before DSH finally ceased (without reoccurring) in the course of treatment, and the median amount of time was 8 weeks. The majority of patients (93.5%) ceased their DSH during treatment, while three patients continued to report DSH. Twenty five percent of the patients ceased their DSH behavior during the first week of treatment. For the remaining patients, cessation of DSH occurred gradually. When comparing early and late DSH responders, there were no differences between groups with regard to age, gender, comorbidity, symptom severity, or treatment outcome. Both groups benefited from DBT, displaying significant reduction in symptom severity and increased functioning.

The low median amount of time before cessation of DSH in this study could be a result of the high focus on the willingness to address this behavior in the pre-treatment phase. This is highlighted by the fact that a quarter of the patients ceased their DSH during the first week of treatment. Despite the fact that DSH often is used as an outcome measure in studies on BPD, few studies report when in the course of treatment self-harm ceases. A study from van Goethem et al. (2015) found that parasuicidal behavior showed a highly variable course although overall decreased during DBT. This is in line with a study by Binks et al. (2006) which reported that self-harm and parasuicide decreased between 6 and 12 months into treatment. Both of these findings are consistent with the heterogeneity in our study.

At the end of treatment, there were three patients (6.5%) who did not cease their DSH behavior. This is a considerable lower proportion than reported in other studies (van Goethem et al., 2015; McCauley et al., 2018) although there were differences in the measurement of the reported cessation of DSH. Other possible explanations could relate to longer duration of treatment and the fact that only treatment completers were included in the analyses. In our study we followed the course of DSH behavior during DBT treatment. McMain et al. (2018) identified a subgroup of patients that although showing an initial rapid response relapsed in the follow-up period. Although self-harmful behavior ceases, there are indications that self-harm impulses can persist (van Goethem et al., 2015). The current study does not include follow-up data, and the extent of relapse in our sample is therefore unclear.

We found no significant difference between the early and late responders in pre-treatment level of symptoms or treatment response. At the end of the treatment, both those who quickly gained control of DSH and those who later gained such skills demonstrated a significant symptom decline. We did, however, find a non-significant trend toward higher symptom severity baseline among early responders. The is in line with Gratz et al. (2014) who found that greater severity in baseline emotion dysregulation and BPD criteria predicted better responses during treatment and follow-up between responders and non-responders in emotion regulation therapy.

The self-harm group was significantly younger than patients without DSH, which is in line with previous research (e.g., Müller et al., 2016). Symptoms of BPD wax and wane, but self-harm seems to decrease over time (Paris and Zweig-Frank, 2001; Fok et al., 2012). The course of BPD may switch from predominantly emotional dysregulation, impulsivity, and suicidality among adolescents, to maladaptive interpersonal functioning in adulthood (Videler et al., 2019). However, the design of the current study cannot address why lower age is associated with DSH. A related limitation is that we did not assess emotional regulation and impulsivity. A possible explanation for the mentioned age association could be due to diminished impulsivity with increasing age (e.g., Stevenson et al., 2003; Steinberg et al., 2008) and that emotion regulation strategies may change with age (Livingstone et al., 2020). It is likely that changes in emotion regulation and impulsivity would be associated with changes in self-harm as emotion dysregulation and impulsivity is associated with self-harm (e.g., Chapman et al., 2008; Terzi et al., 2017; Garofalo et al., 2018). However, it is also important to bear in mind that DSH behaviors may alter in form across age groups.

### Limitations

There were different limitations to the study. The study did not have a control condition. Therefore it is impossible to conclude whether the findings are unique to DBT. Furthermore, the length of treatment varied greatly. Having a set length of treatment could have affected the results. Also, there was no follow-up assessment of self-harming behavior. Another limitation is that dividing patients into early and late responders was done using a median split. Whether or not the 8 week criterion actually represents a meaningful cutoff point for these two groups is somewhat unclear. Finally, the study included several different measures assessing a wide range of symptoms, functioning, and personality. However, none of these differed among patients who ceased self-harm early or late. There could possibly be other factors that do distinguish these groups, like factors related to emotional dysregulation and impulsivity. A larger sample size and addition of other variables would be important for future studies to consider.

### Clinical Implications and Further Research

DBT appear to be an effective treatment for reducing DSH behavior, and discontinuation of self-harming behavior often occurs relatively early in the course of treatment. Increased knowledge about factors associated with DSH response time could help to tailor treatment. Future research should therefore investigate how changes in emotion regulation and impulsiveness is associated with changes in DSH. The most obvious clinical implication from the current study is the fact that self-harming behavior gradually decreases across the first year in treatment. Limiting treatment to a set 12 weeks for instance would likely produce different results. About 50% had terminated their self-harming after 12 weeks of treatment in the current study compared to more than 90% at week 52. One possible reason for the proliferation of response time can be due to individual differences in how long it takes before new adaptive self-regulation skills are learned and incorporated as an alternative to DSH.

A proportion of patients do not cease their DSH during treatment. In our sample, this subgroup was considered too small to analyze statistically, but could be a subject for case studies to understand why some individuals do not respond behaviorally to comprehensive treatment. To further investigate predictors of patient subgroups who do not respond, late respond, or who is vulnerable to relapse of DSH will be of important clinical significance. It would also be of importance to investigate the patient group who is presenting DSH but do not want to initiate treatment, or prematurely drop out of it.

There is need for even larger and more controlled studies of DBT and the relation to cessation of self-harm. There is also need of more follow-up studies to investigate the long term effects of BPD treatments in relation to cessation of self-harm behavior.

## Conclusion

The findings indicate that although the course of DSH cessation in DBT is highly heterogeneous and erratic, the treatment is effective in reducing self-harm in patients with BPD. The time before self-harm behavior ceases is not necessarily related to comorbidity, symptom severity or symptom reduction. This highlights the importance of retaining patients in DBT treatment although they may strive to end their self-harm behavior. Self-harm behavior may be a specific component relatively unrelated to other symptom characteristics.

## Data Availability Statement

The raw data supporting the conclusions of this article will be made available by the authors, without undue reservation.

## Ethics Statement

The studies involving human participants were reviewed and approved by the Norwegian Regional Committees for Medical and Health Research Ethics. The patients/participants provided their written informed consent to participate in this study.

## Author Contributions

YW was responsible for conducting the study and writing the manuscript draft. KH and SS were responsible for statistical analyses and revising the manuscript. All authors contributed to interpreting the results and to conceptualizing and assisting with editing the manuscript into its final form. All authors contributed to the article and approved the submitted version.

## Conflict of Interest

The authors declare that the research was conducted in the absence of any commercial or financial relationships that could be construed as a potential conflict of interest.
